# Optimal Timing for Auditory Brainstem Response After Tympanostomy Tube Placement in Children with Cleft Lip and Palate: A Retrospective Study

**DOI:** 10.3390/children12091243

**Published:** 2025-09-16

**Authors:** Koichiro Oyake, Sei Kobayashi, Tomotaka Shimura, Yasunobu Amari, Ayaka Kise, Naoto Miyoshi, Naomi Imaizumi, Yukiko Inoue, Toshikazu Shimane

**Affiliations:** 1 Department of Otorhinolaryngology, Showa University Fujigaoka Hospital, Yokohama 227-8501, Japan; sei.ent@med.showa-u.ac.jp (S.K.); t-shimura@med.showa-u.ac.jp (T.S.); yasuyasu@med.showa-u.ac.jp (Y.A.); a.kise3031@med.showa-u.ac.jp (A.K.); n-miyoshi@med.showa-u.ac.jp (N.M.); naomi0615@med.showa-u.ac.jp (N.I.); yukiko1013@med.showa-u.ac.jp (Y.I.); 2 Department of Otorhinolaryngology Head & Neck Surgery, Showa University Hospital, Tokyo 142-8666, Japan; shima-tskz@dent.showa-u.ac.jp

**Keywords:** cleft lip and palate, hearing loss, otitis media with effusion, newborn hearing screening, tympanostomy

## Abstract

**Highlights:**

**What are the main findings?**

- ABR wave V thresholds and wave I latencies significantly improved after tympanostomy in CLP children with OME, suggesting that a substantial portion of the hearing loss experienced was conductive and reversible;
- Postoperative ABR assessments, particularly those conducted on or after postoperative day 15, showed better wave V thresholds, with a higher proportion of ears achieving thresholds below 40 dBnHL.

**What are the implications of the main findings?**

- In infants with CLP, ABR testing performed during untreated OME or within the early postoperative period may overestimate hearing loss due to residual middle ear pathology;
- Conducting ABR reassessment approximately 15 days after tympanostomy allows for more reliable interpretation and may help to alleviate early parental anxiety regarding potential hearing impairment.

**Abstract:**

**Objective**: Children with cleft lip and/or palate (CLP) commonly present with otitis media with effusion (OME), with increased referrals for newborn hearing screening (NHS). Auditory brainstem response (ABR) testing with OME may mimic sensorineural hearing loss. This study evaluated NHS and ABR findings on and optimal timing for ABR reassessment after tympanostomy in patients with CLP. **Methods**: We conducted a retrospective study reviewing 271 CLP cases at our institution. The data included the cleft type, NHS results, ABR findings, OME incidence, and tympanostomy rate. Subgroup analyses compared ABR results before and after tympanostomy and via postoperative timing. Statistical comparisons were performed using the Mann–Whitney U test and Fisher’s exact test. **Results**: The NHS referral rate was 14.0%, and the OME incidence was 48.7%. These cases occurred in patients with cleft palate involvement, with an OME prevalence of 73.4%. Tympanostomy was performed in 72.6% of cases. Among 36 ears tested pre- and post-tympanostomy, wave V thresholds improved from 61.67 ± 16.08 to 34.72 ± 6.54 dBnHL (*p* < 0.0001), and wave I latency decreased from 2.27 ± 0.36 to 1.76 ± 0.12 ms (*p* < 0.0001). Postoperative wave V thresholds were significantly better in the ≥15-day group (*p* = 0.037), with 65% (17/26) of ears showing thresholds <40 dBnHL compared to 25% (3/12) in the <15-day group (*p* = 0.035). No timing-related differences were found regarding wave I latency. **Conclusions**: Tympanostomy significantly improved the ABR results in children with CLP and OME. Reassessment on or after postoperative day 15 may yield more accurate results and may help to reduce parental anxiety.

## 1. Introduction

Cleft lip and/or palate (CLP) is one of the most common congenital craniofacial anomalies, occurring in 1–2 cases per 1000 live births in many countries, including Japan [1,2]. CLP may involve the lip, alveolus, palate, or a combination thereof. It results in a wide spectrum of morphological and functional impairments, such as feeding, speech, and hearing difficulties [1,2]. Persistent and treatment-resistant otitis media with effusion (OME) is a frequent auditory complication in children with CLP [3,4]. This condition arises from anatomical disruption of the tensor veli palatini and levator veli palatini muscles, which causes eustachian tube dysfunction [4]. The reported prevalence of OME in children with CLP aged between 2 and 24 months ranges from 76% to 97% [5]. If left untreated, OME may lead to irreversible conductive hearing loss (e.g., tympanic membrane atrophy/scarring or ossicular chain fixation). It may also progress to chronic, adhesive, or cholesteatomatous otitis media [3]. This condition can have potential adverse effects on speech and language development, underscoring the importance of early diagnosis and intervention [6].

In recent years, widespread implementation of newborn hearing screening (NHS) has facilitated the early identification and management of hearing loss [7]. Although the referral rate in the general newborn population is approximately 4% [8], higher rates ranging from 11% to 28% have been reported in children with CLP [7,9]. CLP infants who perform poorly on NHS typically undergo further evaluation with ABR [10]. However, no consensus exists regarding the optimal timing or methods for such assessments and interventions. Most cases of hearing loss in this population are attributable to postnatal-onset OME [7], whereas congenital sensorineural hearing loss is relatively rare [3,11]. Nevertheless, ABR testing in children with CLP and concurrent OME often demonstrates significantly elevated wave V thresholds that mimic sensorineural hearing loss, thereby complicating interpretation [12,13]. Although the high prevalence of OME and increased NHS referral rates in CLP are well recognized, most available data come from Western cohorts. Systematic evaluations linking NHS outcomes with subsequent ABR findings and tympanostomy remain scarce in our region.

Recent empirical studies have reported the effectiveness of tympanostomy in children with CLP, including improvements in postoperative ABR outcomes [13,14,15,16]. However, these studies primarily assessed outcomes several months after surgery rather than focusing on the very early postoperative period. To the best of our knowledge, no previous studies have specifically assessed the optimal time at which clinicians should perform ABR reassessment in CLP infants who initially perform poorly on NHS tests and present with abnormal ABR findings due to OME. Such uncertainty is clinically important, as false-positive ABR findings may result in unnecessary interventions or delayed recognition of true congenital sensorineural hearing loss. Accordingly, this study aimed to retrospectively investigate the incidence of OME and NHS referral rates in children with CLP and to analyze ABR findings to determine the optimal timing for postoperative reassessment and the earliest point at which reliable threshold measurements can be obtained.

## 2. Materials and Methods

### 2.1. Patient Cohort

**Figure 1:**
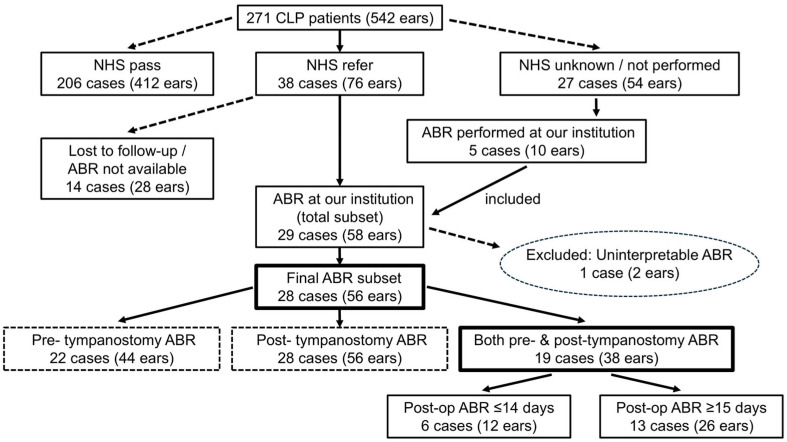
Patient flow diagram showing cohort and subgroup formation. The diagram outlines the overall patient cohort, subsequent inclusion or exclusion, and the formation of subgroups for ABR analyses. CLP: cleft lip and/or palate; NHS: newborn hearing screening; ABR: auditory brainstem response.

A total of 271 CLP patients (542 ears) who visited our center before the age of one year old between April 2017 and August 2021 were included in our study, which was primarily based on a review of clinical records. Figure 1 summarizes the overall patient flow and illustrates how subgroups were defined for subsequent ABR analyses.

### 2.2. NHS and Referral Rates by Cleft Type

The NHS results were obtained during the initial clinical interview based on reports from birth facilities. The outcomes were categorized as bilateral pass, bilateral referral, unilateral referral, or unknown. Refer rates were analyzed for the entire cohort and stratified according to cleft type. The type of screening test used (automated auditory brainstem response [AABR], distortion product otoacoustic emissions [DPOAEs], or transient-evoked otoacoustic emissions [TEOAEs]) varied by birth facility. Although previous large-scale studies have shown that AABR tends to result in lower referral rates than DPOAEs or TEOAEs, the overall clinical value of these tests for NHS is considered comparable when evaluated in terms of cost, sensitivity, specificity, and practical utility [17]. Therefore, in this study, the three screening modalities were treated as equivalent and were not analyzed separately.

### 2.3. ABR

Among the children who received a referral result (unilateral or bilateral) via NHS, those who underwent ABR testing at our institution and whose detailed ABR results were accessible were included in the ABR analysis. In addition, children whose NHS results were unknown or not performed, but who subsequently underwent ABR testing after presentation to our institution, were also included. All ABR tests were performed using click stimuli. For infants younger than 3 months, ABR was generally performed during natural sleep. For those aged 3 months or older, all examinations were conducted under sedation with Triclofos Sodium at a dose of 20–50 mg/kg. As previous reports indicated that Triclofos Sodium sedation does not significantly affect ABR waveform amplitudes, thresholds, or latencies compared with natural sleep [18], both groups were included in the present analysis.

Given the developmental characteristics of ABR and the age distribution of our cohort, we selected wave V threshold and wave I latency as the primary outcome parameters. The wave V threshold is widely used for screening sensorineural hearing loss [19], and it has been further recognized as a useful indicator for identifying sensorineural hearing loss in infants and young children [20]. In this study, a wave V threshold <40 dBnHL was considered within the clinically accepted range and not indicative of hearing loss, while thresholds ≥40 dBnHL were considered suggestive of hearing loss in accordance with the standards used in the general pediatric population [20,21]. This cutoff was uniformly applied across all ages in our cohort because the limited number of ABR cases precluded establishing age-stratified criteria. The prolongation of wave I latency has been reported to be sensitive to the presence of OME [22], and because wave I latency reaches adult values by around 3 months of age [20,23], it was suitable for evaluating the impact of OME in this cohort.

Based on previous evidence that click stimuli at 80 dBnHL yield optimal waveform reproducibility and amplitude [24], wave I latency was measured using 80 dBnHL click stimuli. While few studies have defined specific cutoffs for prolonged wave I latency, several reports involving large numbers of normal-hearing infants indicate that the mean + 2.5 to 3 SD corresponds to approximately 2.00 ms [25,26]. Accordingly, a wave I latency ≥ 2.00 ms was defined as prolonged. Similarly, because a threshold of 80 dBnHL corresponds to the conventional definition of severe hearing loss, ears without a discernible wave V at this level were categorized as experiencing suspected severe hearing loss.

The ABR results were compared between the pre- and post-tympanostomy groups based on the timing of the test in relation to tympanostomy tube placement. We also conducted subgroup analyses to assess whether the timing of tympanostomy influenced ABR outcomes.

As an additional analysis, post-tympanostomy ABR cases were classified into two subgroups based on the timing of the test (≤14 days and ≥15 days after surgery), as shown in Figure 1. This two-week threshold was based on a prior study that demonstrated the normalization of middle ear mucosal inflammation and ultrastructure approximately two weeks after tympanostomy [27]. Wave V thresholds and wave I latencies were compared between the two subgroups.

To determine the most appropriate cutoff values for distinguishing between the ≤14-day and ≥15-day groups, a series of candidate thresholds for wave V and wave I latency were explored. For each candidate cutoff, 2 × 2 contingency tables were generated and Fisher’s exact test was applied to identify statistically significant group differences. The cutoff values yielding the smallest *p*-values were considered to be the most discriminative.

### 2.4. Assessment of OME Prevalence and Tympanostomy Tube Placement

We assessed the prevalence of OME, rate of tympanostomy tube placement, timing of tube insertion, and type of tympanostomy tube used across the study cohort. Otoscopic examinations were performed by two experienced pediatric otolaryngologists (K.O. and Y.A.), and OME was defined as the persistent presence of effusion for ≥3 months during the observation period.

Tympanostomy tubes in children with CLP are placed concurrently with either cheiloplasty at 3–4 months of age or palatoplasty at 12–15 months of age according to the standard clinical consensus [14]. From a language development perspective, timely intervention for OME is particularly important in children aged between 12 and 18 months [6,14]. However, the optimal timing of tympanostomy, whether during lip or palate surgery or at a specific age, remains a subject of ongoing debate [28].

At our institution, the indications for tympanostomy tube placement are determined as follows:

During cheiloplasty: Tubes are placed at the time of cheiloplasty if all the following conditions are met: (1) unilateral or bilateral referral for NHS; (2) marked tympanic membrane retraction observed both at the initial otolaryngology consultation and immediately before surgery; and (3) strong parental concern regarding OME or hearing loss, with a desire for early intervention. Tympanostomy tube placement is permitted in such cases, even if the OME duration is less than three months.

Tubes are placed at the time of palatoplasty if OME was documented according to the above definition during the observation period and effusion was still evident immediately before surgery.

Other timing: In cases in which tube placement cannot be performed during lip or palate surgery for clinical or logistical reasons, tympanostomy is performed either under local anesthesia in the outpatient setting or under general anesthesia by the otolaryngology team alone. For patients with isolated cleft palate (i.e., incomplete cleft palate without associated cleft lip or alveolus, including submucous cleft palate), tympanostomy tube placement is also performed under local anesthesia when all three criteria described under “During cheiloplasty” are met, even outside the time at which primary lip or palate surgery is performed.

For tube selection, a Paparella type 2 tympanostomy tube is used as the standard because of its lower extrusion rate [29]. However, in cases of severe external auditory canal stenosis or reduced middle ear space due to mucosal hypertrophy, Paparella type 1 tubes are used. Regardless of the laterality of the NHS, based on the results or OME findings, all tympanostomy procedures are performed bilaterally.

### 2.5. Statistical Analysis

All the participants in this study were pediatric patients. For comparisons of continuous variables, such as the ABR wave V thresholds and wave I latencies, between the two groups, the Mann–Whitney U test was applied, as none of the groups satisfied the assumptions of normality. Fisher’s exact test was used to compare categorical data (e.g., distributions based on cutoff values and post-tympanostomy ABR timing). All statistical analyses were performed using EZR (Saitama Medical Center, Jichi Medical University, Saitama, Japan), a graphical user interface for R (version 4.3.1; R Foundation for Statistical Computing, Vienna, Austria). Statistical significance was defined as a *p*-value < 0.05.

## 3. Results

### 3.1. Overall Cohort Characteristics

In total, 271 children (542 ears) were included in this study. The cohort comprised 176 males (352 ears) and 95 females (190 ears), with a mean age of 2.97 ± 3.35 months at initial presentation. Based on cleft classification, 32 patients had cleft lip; 60 had cleft lip and alveolus; 114 had cleft lip, alveolus, and palate; and 65 had isolated cleft palate.

Associated syndromes or congenital anomalies included Pierre Robin sequence (*n* = 5), trisomy 21 (*n* = 3), Kabuki syndrome (*n* = 1), Dandy–Walker syndrome (*n* = 1), 22q11.2 deletion syndrome (*n* = 1), Treacher Collins syndrome (*n* = 1), ectodermal dysplasia (*n* = 1), and monosomy of chromosome 4 (*n* = 1). All patients had bilateral tympanic membrane visibility; no patients were excluded because of an inability to assess the tympanic membranes.

The follow-up period for each patient was defined based on the available clinical records and was not uniform across the cohort. The reviewed period was selected individually for each case depending on the clinical course and relevance of the data to the study objectives.

For patients who underwent tympanostomy, the review period was extended from the initial visit to the time of surgery and, when applicable, to the time of postoperative ABR reassessment. Tympanostomy was generally performed at the time of cheiloplasty (approximately 3–4 months of age) or palatoplasty (approximately 12–15 months of age) depending on the surgical schedule.

In patients who did not undergo tympanostomy, such as those with bilateral pass noted during NHS or with only transient OME findings, the record review covered at least the period between the initial cleft surgery and the time around palatoplasty. Some patients continued to receive otologic care beyond the period analyzed in this study; however, as the reviewed duration was tailored to each patient, a standardized endpoint could not be applied across the cohort. As the follow-up length was not a primary analytical variable in this study, we did not calculate summary statistics such as the mean or median duration.

### 3.2. NHS Results and Referral Rates by Cleft Type

Among the entire cohort, the overall ‘referral’ rate for NHS was 14.0% (38 of 271 cases), including 6.3% with unilateral referrals and 7.7% with bilateral referral results. The details of the cleft types are summarized in Table 1. All patients with cleft lip or cleft lip and alveolus alone (excluding those with unknown or unperformed NHS results) showed bilateral pass results, with no confirmed referred cases in these subgroups. In contrast, all the referred cases occurred in children with palatal involvement. Among the 179 patients with any form of cleft palate, the referral rate was 21.2% (38 of 179 cases).

Owing to the uneven distribution of cleft types and the limited number of cases in certain subgroups, performing the statistical analysis of NHS referral rates by specific cleft subtypes beyond the presence or absence of palatal involvement was not feasible.

### 3.3. Prevalence of OME and Tympanostomy Tube Placement in the Overall Cohort

Among the entire cohort of 271 patients, the overall prevalence of OME was 48.7% (132/271), and the tympanostomy tube placement rate was 47.9% (130/271). All patients who developed OME or underwent tympanostomy had cleft palate involvement. None of the patients with cleft lip or cleft lip and alveolus alone developed OME during the review period or required tympanostomy.

Focusing specifically on 179 patients with cleft palate (with or without lip/alveolar involvement), the OME prevalence was 73.4% (132/179), and nearly all affected patients underwent tympanostomy tube placement (72.6%; 130/179). The two OME-positive patients who did not undergo tympanostomy experienced spontaneous resolution of effusion following either cheiloplasty or soft palate adhesion repair and maintained a healthy tympanic membrane status thereafter.

Among the 130 tympanostomy cases, 50 patients (100 ears) received tubes at the time of cheiloplasty, whereas 80 patients (160 ears) underwent tube placement during palatoplasty. A total of 260 ears received tympanostomy tubes, with long-term tubes (Paparella type II) used in 245 ears (94.2%) and short-term tubes (Paparella type I) used in 15 ears (5.8%). There were no cases in which tube placement was deemed infeasible, nor were there any unilateral insertions.

### 3.4. Evaluation of ABR Findings in Relation to Tympanostomy Timing

#### 3.4.1. Overview of ABR Data Inclusion

ABR testing was performed in patients who showed unilateral or bilateral referral results during NHS. In addition, five patients (10 ears) whose NHS results were either unknown or not performed also underwent ABR at our institution, and their data were included in the analysis. Of the 38 cases confirmed to have received a referral result on NHS, 14 were not followed up with, either because ABR had been performed at outside institutions and the results could not be obtained or because follow-up was discontinued when patients stopped attending our clinic. Among the 27 cases with unknown NHS results, only 5 underwent ABR testing at our institution, while the remaining cases could not be followed up with due to the unavailability of detailed information or the discontinuation of visits. ABR data were initially available for 29 patients (58 ears). However, one patient (two ears) with chromosome 4 monosomy, who required mechanical ventilation and exhibited uninterpretable bilateral waveforms due to respiratory artifacts, was excluded. Thus, 28 patients (56 ears) were ultimately included in the analysis. ABR testing was conducted either before or after tympanostomy, or at both time points. Specifically, 22 patients (44 ears) underwent ABR testing before tympanostomy, 28 (56 ears) underwent ABR testing after tympanostomy, and 19 (38 ears) underwent ABR assessments both before and after surgery. However, these groups were not mutually exclusive. Among the patients who underwent postoperative ABR retesting after tympanostomy, 67.9% were tested within 30 days of surgery. In many of the remaining cases, testing was initially scheduled within the 30-day window; however, owing to the patient’s inability to sleep at the time of testing, the procedure had to be rescheduled and was ultimately conducted on or after postoperative day 31. A small subset of patients underwent ABR retesting > 100 days postoperatively, although the reasons for such prolonged delays are not well documented.

#### 3.4.2. ABR Findings Before Tympanostomy

Among the 22 patients (44 ears) who underwent ABR before tympanostomy, the mean age at testing was 2.86 ± 1.96 months (range: 0–7 months). Wave V thresholds were interpretable for all ears. However, wave I was not identifiable in 12 ears from six patients, and these ears were excluded from the latency analysis. Of these 22 patients, 4 (8 ears) underwent ABR during natural sleep, whereas all the remaining cases were conducted under sedation with Triclofos Sodium. The mean wave V threshold was 59.55 ± 17.08 dBnHL (range: 30–105 dBnHL), and the mean wave I latency was 2.19 ± 0.40 ms (range: 1.54–2.94 ms). Based on the study criterion, 40 ears (91.0%) had thresholds ≥40 dBnHL, indicating abnormal values. A total of 21 ears (47.7%) showed prolonged wave I latency (≥2.00 ms). Five ears in three patients had wave V thresholds of 80 dB nHL or higher. Although these patients were provisionally diagnosed with suspected severe congenital hearing loss, all three were later determined to have normal hearing.

#### 3.4.3. ABR Findings After Tympanostomy

Among the 28 patients (56 ears) who underwent ABR testing after tympanostomy, the mean age at testing was 10.55 ± 6.78 months (range: 4–35 months). One patient had undetectable wave I in both ears, and their ears were excluded from the relevant analyses. All postoperative ABR assessments were conducted under sedation with Triclofos Sodium. The mean wave V threshold was 34.64 ± 8.94 dBnHL (range: 20–70 dBnHL), and the mean wave I latency was 1.75 ± 0.19 ms (range: 1.43–2.58 ms). According to the same criterion, 24 ears (42.8%) exhibited thresholds ≥40 dBnHL. Only three ears (5.2%) exhibited prolonged wave I latency. No ears exhibited wave V thresholds of 80 dBnHL or higher after tympanostomy. Although 42.8% of ears still demonstrated wave V thresholds ≥40 dBnHL postoperatively, all were later confirmed not to have permanent sensorineural hearing loss. The oldest patient in this group (35 months old) was lost to follow-up despite bilateral NHS referral at birth and presented again only after a prolonged period of neglect. Tympanostomy and ABR were performed at the time of re-presentation.

#### 3.4.4. Comparison of ABR Before and After Tympanostomy

**Figure 2:**
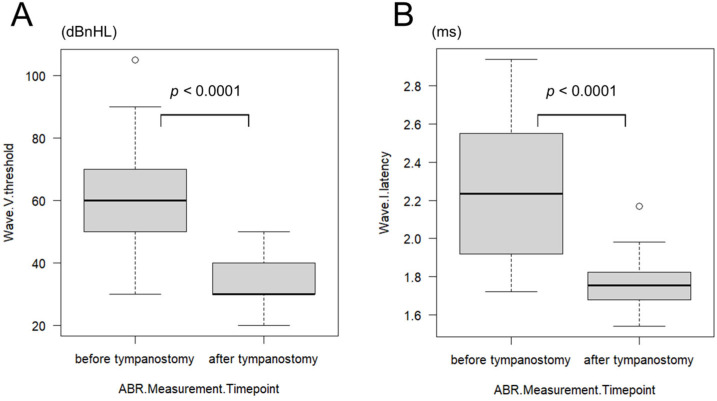
A comparison of wave V thresholds (**A**) and wave I latencies (**B**) before and after tympanostomy. Both wave V thresholds and wave I latencies showed significant improvement after tympanostomy (*p* < 0.0001). ABR: auditory brainstem response.

In the subset of 19 patients (38 ears) who underwent ABR testing before and after tympanostomy, wave V thresholds were interpretable in all ears. However, wave I latency was unmeasurable in 10 ears of five patients, which were excluded from the latency analysis. The mean age at preoperative ABR was 2.50 ± 1.98 months (range: 0–7 months). The mean wave V threshold was 61.67 ± 16.08 dBnHL (range: 30–105 dBnHL), and wave I latency was 2.27 ± 0.36 ms (range: 1.72–2.94 ms). At postoperative ABR, the mean age was 10.39 ± 7.35 months (range: 4–35 months), with a mean wave V threshold of 34.72 ± 6.54 dBnHL (range: 20–50 dBnHL) and a mean wave I latency of 1.76 ± 0.12 ms (range: 1.54–2.17 ms). Both wave V thresholds and wave I latencies showed statistically significant improvements after tympanostomy (*p* < 0.0001; wave V: HL diff = 25 dBnHL [95% CI 25–30], r = 0.87; wave I: HL diff = 0.53 ms [95% CI 0.34–0.69], r = 0.85) (Figure 2A,B).

Subgroup analysis based on the timing of tympanostomy was also performed. In the group that underwent tympanostomy at the time of cheiloplasty (*n* = 10 patients, 20 ears), the mean preoperative wave V threshold was 63.50 ± 18.07 dBnHL (range: 30–105 dBnHL), improving to 35.00 ± 9.29 dBnHL (range: 20–70 dBnHL) after surgery. The mean wave I latency improved from 2.21 ± 0.39 ms (range: 1.72–2.94 ms) to 1.77 ± 0.23 ms (range: 1.43–2.58 ms). In the group that underwent tympanostomy at the time of palatoplasty (*n* = 4 patients, 8 ears), the mean preoperative wave V threshold was 51.25 ± 8.35 dBnHL (range: 40–60 dBnHL), improving to 30.71 ± 6.16 dBnHL (range: 20–40 dBnHL) postoperatively. The mean wave I latency improved from 2.28 ± 0.34 ms (range: 1.76–2.84 ms) to 1.72 ± 0.09 ms (range: 1.55–1.85 ms). In both subgroups, significant improvements were observed in wave V thresholds and wave I latencies after tympanostomy. The preoperative wave V threshold was significantly higher in the cheiloplasty group than in the palatoplasty group (*p* = 0.040). However, the magnitude of improvement in both wave V threshold and wave I latency did not differ significantly between the two groups. Four additional cases (eight ears) in which tympanostomy was performed at non-standard times were excluded from this analysis.

#### 3.4.5. Effect of ABR Timing After Tympanostomy

**Figure 3:**
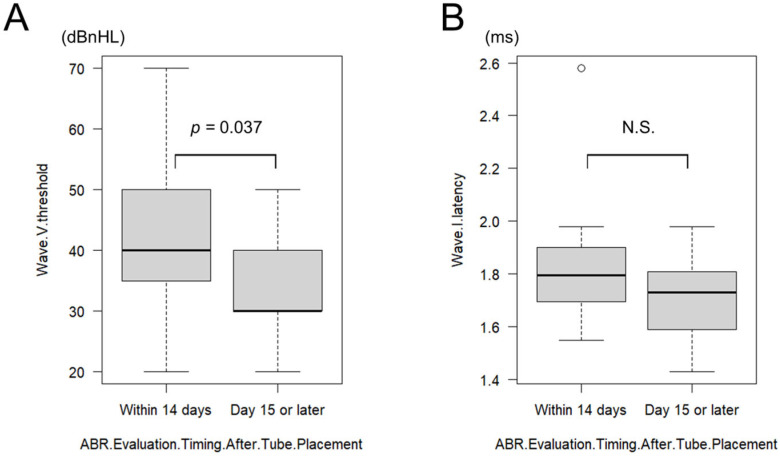
A comparison of wave V thresholds and wave I latencies based on the timing of ABR re-evaluation after tympanostomy: (**A**) Wave V thresholds were significantly better in the 15-day group than in the 14-day group (*p* = 0.037). (**B**) In contrast, there was no significant difference in Wave I latency between the two groups. ABR: auditory brainstem response; N.S.: not significant (*p* > 0.05).

The effect of postoperative ABR timing was further examined. To reduce the potential confounding effect of prolonged recovery, a supplementary analysis was performed that included only ears with postoperative ABR conducted within 30 days of tympanostomy. Of the patients who underwent ABR testing after tympanostomy, 6 patients (12 ears) were tested within 14 days postoperatively (mean: 9.33 ± 2.67 days; range: 6–14 days) and 13 patients (26 ears) were tested on or after postoperative day 15 (mean: 21.84 ± 5.60 days; range: 15–30 days). In the ≤14-day group, the mean wave V threshold was 40.83 ± 13.79 dBnHL (range: 20–70 dB), and the mean wave I latency was 1.84 ± 0.26 ms (range: 1.55–2.58 ms). In the ≥15-day group, the mean wave V threshold was 33.07 ± 6.79 dBnHL (range: 20–50 dB), and the mean wave I latency was 1.70 ± 0.13 ms (range: 1.43–1.98 ms). Wave V thresholds were significantly better in the ≥15-day group (*p* = 0.037, HL diff = 10 dBnHL [95% CI 0–10], r = 0.34) (Figure 3A). Although wave I latency tended to be shorter in the ≥15-day group, the difference was not statistically significant (*p* = 0.116, HL diff = 0.09 ms [95% CI −0.02–0.21], r = 0.26) (Figure 3B).

An additional analysis was performed to identify the optimal cutoff values for differentiating ABR outcomes based on the timing of the postoperative assessment. For wave V threshold, Fisher’s exact test showed that a cutoff value of 40 dBnHL yielded the strongest separation between the ≤14-day and ≥15-day groups (*p* = 0.035), suggesting clinical utility for this threshold (Table 2). For wave I latency, a cutoff of 1.96 ms showed the greatest separation trend, although statistical significance was not reached (*p* = 0.084) (Table 3).

#### 3.4.6. Other Findings

Finally, based on supplementary data, ABR testing was performed in nine patients (18 ears) who had bilateral pass results from NHS. Among these, four patients (8 ears) underwent ABR before tympanostomy, with a mean wave V threshold of 58.75 ± 19.59 dBnHL (range: 30–80 dBnHL) and a mean wave I latency of 1.95 ± 0.42 ms (range: 1.48–2.47 ms). The remaining seven patients (14 ears) underwent ABR after tympanostomy, with a mean wave V threshold of 30.00 ± 6.79 dBnHL (range: 20–40 dBnHL) and a mean wave I latency of 1.61 ± 0.13 ms (range: 1.38–1.88 ms). Owing to the small sample size within these subgroups, statistical comparisons with other groups were not performed.

## 4. Discussion

In this study, we investigated a large cohort of 271 children with CLP, totaling 542 ears, to assess multiple clinical parameters, including the NHS referral rate, the prevalence of OME, and the rate of tympanostomy tube placement. Of these, a subset of 38 ears was evaluable for pre- and postoperative ABR reassessment and served as the basis for our analysis of timing. Among the various findings, a particularly noteworthy result is the observation that the middle ear environment tends to improve relatively early after tympanostomy, with postoperative day 15 appearing to mark a turning point. ABR findings are more likely to accurately reflect this improvement when reassessed around that time. To the best of our knowledge, no prior studies have specifically addressed the question of how long clinicians should wait before repeating ABR in children with CLP who initially failed NHS and showed abnormal ABR results due to OME. Furthermore, by enabling the early acquisition of reliable test results, this approach may facilitate more informed treatment decisions and, as a consequence, help to reduce parental anxiety among families of children with CLP.

Our cohort’s overall NHS referral rate and the high prevalence of OME in patients with cleft palate were consistent with those of previous studies [5,7,9], reaffirming that the presence of palatal involvement is a key factor influencing early otologic outcomes [30]. Although not the primary focus of this study, these findings align with the existing literature and support the clinical relevance of cleft-type stratification in early screening and intervention strategies.

Based on our findings, it is important to consider the anatomical and physiological characteristics of OME in children with CLP when interpreting suboptimal ABR results prior to performing tympanostomy. Previous studies have suggested that OME in CLP is not only persistent but also often associated with highly viscous middle ear effusion and structural abnormalities in the middle ear [31]. Chronic negative pressure and long-standing effusion may induce sustained alterations in the stiffness and mass of the middle ear conduction system, which could unevenly affect ABR thresholds compared with non-CLP OME cases [32,33].

Unlike transient OME-related conductive hearing loss typically observed in otherwise healthy infants, CLP-associated OME may be accompanied by fluctuating and unstable impedance characteristics due to abnormal eustachian tube function and the underdevelopment of the associated musculature [32]. Such irregularities may distort the temporal fidelity of auditory signal transmission, even when the stimulus intensity is controlled, resulting in sustained wave I delay and falsely increased wave V thresholds [13,34]. This may partially explain why some patients in our cohort demonstrated poor ABR findings before tympanostomy, including wave V thresholds exceeding 80 dBnHL, despite being confirmed to have normal hearing.

Another consideration is that ABR is often performed very early in life, including during the neonatal period and typically before three months of age, in infants who perform poorly on NHS [10,35]. At this early stage, ongoing myelination and immature synaptic synchrony in the auditory pathway may inherently limit waveform detectability, particularly for wave I. Furthermore, small delays or measurement inconsistencies during this early period may be interpreted as abnormal, particularly in the presence of concurrent middle ear pathology [20].

Our findings are consistent with the understanding that improvements in wave V threshold and wave I latency following tympanostomy are largely attributable to changes in middle ear status rather than true sensorineural deficits. Importantly, this improvement was consistently observed in both the early (cheiloplasty-associated) and later (palatoplasty-associated) tympanostomy groups, supporting the reversibility of ABR abnormalities in the presence of OME and reinforcing the clinical value of post-tympanostomy ABR reassessment in this population. These findings also support the notion that the true prevalence of congenital sensorineural hearing loss among infants with CLP with initial NHS referral and abnormal ABR results is likely low.

Notably, even among infants with bilateral NHS pass results, poor ABR findings were observed when testing was performed early in life without prior tympanostomy, with wave V thresholds as high as 80 dBnHL recorded in some cases. This further underscores the limited reliability of ABR in infants with CLP and unaddressed middle ear effusion.

In the subgroup analysis based on the timing of ABR reassessment, patients tested on or after postoperative day 15 showed significantly better ABR outcomes than those tested within 14 days (*p* = 0.037). This finding aligns with the histopathological observations reported by Elkholy et al. (2021) [27], suggesting the near-complete recovery of the middle ear mucosa within approximately two weeks of tympanostomy tube placement. Although that study did not involve children with CLP and it remains debatable whether postoperative changes in the middle ear environment can be directly extrapolated to this population, it nevertheless provides supportive evidence for the biological plausibility of a two-week recovery period. The efficacy of tympanostomy for OME in children with CLP has been demonstrated in previous reports [14,15,16]; however, those evaluations were generally conducted several months after surgery, and little has been said in the literature regarding improvements on a week-to-week basis. To the best of our knowledge, no prior studies have provided detailed information on the week-to-week changes in the middle ear environment during the early postoperative period after tympanostomy in CLP patients. Therefore, our observation that ABR results become more reliable from postoperative day 15 or later represents novel short-term evidence that complements the existing literature and aligns with the expected resolution of middle ear pathology within this timeframe. From a clinical perspective, specifying this interval provides a practical guide for balancing the need for timely reassessment with the goal of obtaining reliable test outcomes. Nevertheless, it should be noted that earlier reassessment may still be warranted in selected cases, such as infants with suspected profound sensorineural hearing loss, those with syndromic risk factors, or when urgent parental concerns necessitate prompt clarification. This insight may allow for more informed hearing re-evaluation and clinical decision-making in infants with CLP.

Interestingly, we found that applying a wave V threshold cutoff of 40 dBnHL significantly differentiated outcomes between the ≤14-day and ≥15-day groups (*p* = 0.035) (Table 2). The 40 dBnHL threshold has long been recognized as a useful benchmark for distinguishing normal hearing from abnormalities in pediatric populations [20,21]. Our findings suggest that it may also serve as a practical clinical reference point for interpreting post-tympanostomy ABR in infants with CLP. Its application may aid in identifying children who require further audiological monitoring or early speech and language therapy interventions.

In contrast, wave I latency did not show a clearly defined cutoff in this context. This may be due to the inherently lower waveform reproducibility and robustness of wave I compared to wave V [24,36]. Although our study was not designed to investigate these issues in depth, future studies incorporating refined ABR protocols may further clarify these subtle distinctions.

The findings of this study provide important implications for supporting families of infants with CLP. Although it is widely recognized in clinical practice that hearing loss in this population is usually a natural occurrence, our data further support the idea that even markedly abnormal ABR results in the presence of OME do not necessarily indicate permanent sensorineural hearing loss. This highlights the value of post-tympanostomy ABR reassessment at an appropriate time and supports reassurance strategies for families, particularly when retesting is performed after postoperative day 15.

This study has several limitations that must be acknowledged. It was retrospective in nature, which carries inherent constraints in standardizing testing conditions and the follow-up process. Although we confirmed that only a small number of patients underwent ABR during natural sleep and that their results did not differ significantly from those obtained under sedation, complete uniformity of testing conditions could not be ensured. In addition, potential bias arising from changes or delays in examination scheduling due to various factors could not be ruled out. The number of cases with early (≤14 days) postoperative reassessment was limited, which may have reduced the statistical robustness of subgroup comparisons. In addition, some patients were lost to follow-up or had missing ABR data despite referral for newborn hearing screening, which may have influenced the findings. Long-term ABR follow-up data were not consistently available across subgroups, limiting our ability to assess sustained hearing outcomes. Furthermore, the criteria for tympanostomy tube placement were influenced by physician judgment and parental preference, raising the possibility of selection bias; indeed, preoperative wave V thresholds were significantly higher in the early-intervention group, suggesting that cases with higher preoperative thresholds may have been preferentially treated earlier. Because the number of cases with available ABR data was limited, age-stratified normative criteria could not be applied, and a uniform threshold was used across all age groups. Therefore, it should be noted that developmental variability in ABR parameters, particularly during the first few months of life, may have influenced the interpretation of the results. Finally, long-term outcomes such as the recurrence of OME, tube extrusion, or persistent perforation were not evaluated, as our follow-up procedures in this study did not allow for a standardized and reliable assessment of these endpoints. Moreover, given that only 28 cases had analyzable postoperative ABR data, we were unable to further stratify outcomes by cleft subtype or palatoplasty technique, leaving open the possibility that middle ear recovery trajectories might differ according to cleft severity, surgical method, and timing. Nevertheless, the consistent patterns observed across multiple subgroup analyses support the reliability of our main findings.

## 5. Conclusions

Our study demonstrated that OME can significantly distort ABR parameters in infants with CLP, though accurate hearing assessment becomes feasible after tympanostomy, particularly when reassessment is delayed for at least 15 days postoperatively. Incorporating this timeframe into clinical routines may enhance the accuracy of hearing loss diagnoses and help to alleviate parental concerns, thereby enabling more effective care pathways for CLP cases. Nonetheless, earlier reassessment may still be warranted in selected high-risk situations, and clinical judgment remains essential.

## Figures and Tables

**Table 1:** Results of newborn hearing screening by cleft type.

Cleft Type	*n* (Cases)	Bilateral Pass	Unilateral Referral	Bilateral Refer	Unknown/Not Performed
[No palatal involvement]					
Cleft lip	32	30 (93.8%)	0 (0%)	0 (0%)	2 (6.3%)
Cleft lip and alveolus	60	55 (91.7%)	0 (0%)	0 (0%)	5 (8.3%)
Subtotal	92	85 (92.4%)	0 (0%)	0 (0%)	7 (7.6%)
[Palatal involvement]					
Cleft lip and palate	114	76 (66.7%)	13 (11.4%)	19 (16.7%)	6 (5.3%)
Isolated hard palate cleft	41	29 (70.7%)	3 (7.3%)	0 (0%)	9 (22.0%)
Soft palate cleft	18	13 (72.2%)	1 (5.6%)	2 (11.1%)	2 (11.1%)
Submucous cleft palate	6	3 (50.0%)	0 (0%)	0 (0%)	3 (50.0%)
Subtotal	179	121 (67.6%)	17 (9.5%)	21 (11.7%)	20 (11.2%)
Total	271	206 (76.0%)	17 (6.3%)	21 (7.7%)	27 (10.0%)

**Table 2:** The relationship between ABR evaluation timing after tube placement and the wave V threshold.

[Evaluation Timing of ABR]	Wave V Threshold <40 dBnHL	Wave V Threshold ≥40 dBnHL	Total
Within 14 days after tube placement	3 ears	9 ears	12 ears
Day 15 or later	17 ears	9 ears	26 ears
Total	20 ears	18 ears	38 ears

ABR = auditory brainstem response.

**Table 3:** The relationship between ABR evaluation timing after tube placement and wave I latency.

[Evaluation Timing of ABR]	Wave I Latency <1.96 ms	Wave I Latency ≥1.96 ms	Total
Within 14 days after tube placement	9 ears	3 ears	12 ears
Day 15 or later	25 ears	1 ear	26 ears
Total	34 ears	4 ears	38 ears

ABR = auditory brainstem response.

## Data Availability

Data supporting the findings of this study are not publicly available due to privacy concerns. De-identified data may be shared by the corresponding author upon reasonable request and subject to institutional approval.

## Abbreviations

The following abbreviations are used in this manuscript:

NHS	Newborn hearing screening
ABR	Auditory brainstem response
CLP	Cleft lip and/or palate
OME	Otitis media with effusion
